# A Novel Steroidogenic Action of Anti-Müllerian Hormone in Teleosts: Evidence from the European Sea Bass Male (*Dicentrarchus labrax*)

**DOI:** 10.3390/ijms26157554

**Published:** 2025-08-05

**Authors:** Alessia Mascoli, Cinta Zapater, Soledad Ibañez, Mateus Contar Adolfi, Manfred Schartl, Ana Gómez

**Affiliations:** 1 Department of Fish Physiology and Biotechnology, Instituto de Acuicultura Torre de la Sal, Consejo Superior de Investigaciones Científicas (CSIC), Torre la Sal, 12595 Castellón, Spain; alessia.mascoli@iats.csic.es (A.M.); cinta.zapater@csic.es (C.Z.); sole@iats.csic.es (S.I.); 2 Physiological Chemistry, Biocenter, University of Würzburg, 97074 Würzburg, Germany; mateusadolfi@gmail.com; 3 Institute of Pathology, University of Würzburg, 97080 Würzburg, Germany; phch1@biozentrum.uni-wuerzburg.de; 4 Institute for Molecular Life Sciences/*Xiphophorus* Genetic Stock Center, Texas State University, San Marcos, TX 78666, USA; 5 Research Department for Limnology, University of Innsbruck, 5310 Mondsee, Austria

**Keywords:** teleost, fish reproduction, testis, cAMP/PKA pathway, Amhr2

## Abstract

The Anti-Müllerian hormone (AMH) is widely recognized for promoting Müllerian duct regression in higher vertebrates and regulating key reproductive functions like steroidogenesis, folliculogenesis, and Leydig cell development. In teleost fish, which lack Müllerian ducts, Amh primarily influences male reproductive functions, including sex determination, testis differentiation, and germ cell proliferation. In adult fish, Amh supports gonad development and spermatogenesis, but its role in teleost gonadal physiology remains largely underexplored. This study reveals a novel steroidogenic function in the European sea bass (*Dicentrarchus labrax*) using *in vitro* testis culture, *in vivo* plasmid injection, and cell-based transactivation assays. The Amh-induced significant increase in androgen levels was also confirmed in Japanese medaka (*Oryzias latipes*) treated with recombinant sea bass Amh. Beyond activating the canonical Smad pathway, Amh also triggered the cAMP/PKA signalling pathway via its cognate type II receptor, Amhr2. Inhibitors of these pathways independently and synergistically counteracted Amh-induced CRE-Luc activity, indicating pathway crosstalk. Moreover, inhibition of the cAMP pathway suppressed Amh-induced androgen production in testis cultures, emphasizing the crucial role of protein kinase A in mediating Amh steroidogenic action. These findings uncover a novel steroidogenic function of Amh in teleosts and highlight its broader role in male reproductive physiology.

## 1. Introduction

Anti-Müllerian hormone (AMH) is a glycoprotein hormone of the Transforming Growth Factor-β (TGF-β) superfamily, named in the 1940s after its role in inducing Müllerian duct regression in rabbit fetuses, thereby promoting male sex differentiation [1]. Extensive research has since revealed its diverse reproductive functions in humans and higher vertebrates. Human AMH is synthesized as a pre-pro-protein, and further processed through proteolytic cleavage by specific proteases to produce the C-terminal mature form [2]. Dimers of this active form bind specifically and with high affinity to the type II receptor (anti-Müllerian hormone type II receptor, AMHR2), subsequently recruiting and phosphorylating a type I receptor (activin receptor-like kinases, ALK2, ALK3, or ALK6), which in turn phosphorylates cytoplasmatic small mother against decapentaplegic (SMAD) proteins (SMAD1, SMAD 5, or SMAD 8). Finally, activated SMADs interact with the common SMAD4 and translocate to the nucleus to regulate target gene transcription, thus completing the canonical SMAD signalling pathway [3].

In addition to inducing regression of the Müllerian ducts in male mammals, AMH exerts other functions in both sexes. In male mice, it is synthetized by Sertoli cells and is present at high levels in the testis from embryogenic stages to puberty, after which it strongly decreases [4]. Several studies have demonstrated that AMH modulates steroidogenesis by antagonizing gonadotropin action. Elevated AMH levels of this hormone impair luteinizing hormone (LH)-induced testosterone production, modulating androgen biosynthesis during Leydig cell development. In the fetal mouse testis, AMH directly targets steroidogenic cells, inhibiting their proliferation and preventing the differentiation of mesenchymal precursor into Leydig cells [5,6]. In female mice, AMH is produced by granulosa cells mainly in pre-antral and small antral follicles, with expression increasing from infantile period to puberty and remaining high until menopause, when it declines due to follicle pool depletion [4]. AMH plays a central role in folliculogenesis by directly inhibiting primordial follicle recruitment—thus preventing premature oocyte depletion [7,8,9] and indirectly suppressing follicle-stimulating hormone (FSH)-driven follicular growth.

These findings underline the autocrine and paracrine role of AMH in the regulation of reproductive mechanisms in the gonad itself, without excluding its action at a broader level on the Brain–Pituitary–Gonad (BPG) axis, since some studies in mammals reported the presence of AMH and AMHR2 mRNA in gonadotropin-releasing hormone (GnRH) neurons in several brain regions and in the pituitary gland [10,11,12]. However, much is still to be investigated in order to clarify the role of AMH in this complex regulatory system, where numerous players interact and none operates in isolation.

The involvement of AMH in several aspects of reproduction and the enormous variety of reproductive systems that evolved among vertebrates suggest that the ancestral role of AMH is something other than Müllerian duct regression [13]. Teleosts, which lack Müllerian ducts, possess the mammalian orthologue of *amh*. Since its first identification in the Japanese eel (*Anguilla japonica*) [14], this hormone has been found in numerous fish species, including Japanese flounder (*Paralichthys olivaceus*) [15], zebrafish (*Danio rerio*) [16], medaka (*Oryzias latipes*) [17], European sea bass (*Dicentrarchus labrax*) [18], rainbow trout (*Oncorhynchus mykiss*) [19], Nile tilapia (*Oreochromis niloticus*) [20], and others [21,22,23,24]. Despite being present in both sexes, Amh appears to have a more prominent function in males, but with species-specific functional variations. In some species, *amh* is involved in sex determination [25,26,27] and listed among the master sex-determining genes initiating male development [28,29,30]. During juvenile development and gonadal differentiation, strong expression of *amh* is associated with testis differentiation [15,21,31], as well as in species exhibiting temperature-induced masculinization [32]. Several studies have demonstrated the regulation of germ cell proliferation exerted by *amh*. In medaka embryos of both sexes, Amh induces germ cell proliferation, immediately after primordial germ cells (PGCs) reach the gonadal primordium [33], while subsequently it inhibits mitotically active self-renewing germ cells during early gonad differentiation. When the balance between germ cell populations is altered by mutations in the *amh*/*amhr2* system, hyperproliferation of germ cells occurs leading to male-to-female sex reversal with hypertrophic gonads [34], as seen in the medaka *amhr2* mutants and *amh* mutants of zebrafish [35,36] and Nile tilapia [37]. These findings highlight the key role of the hormone in inhibiting spermatogonial proliferation and differentiation, and also in maintaining germ cell quiescence in sex reversal and hermaphroditic species [13,38,39,40,41].

In adult fish testes, Amh (transcript and protein) are found in Sertoli cells surrounding germ cells, mostly undifferentiated [15,40,42], suggesting a role in testicular growth, spermatogenesis, and possible interaction with gonadotropin signalling. *In vivo* and *in vitro* studies in zebrafish have shown that Amh counteracts Fsh activity, inhibiting androgen production, specifically 11-ketotestosterone (11-KT), thereby maintaining spermatogonia in an undifferentiated state [43,44]. Complex feedback dynamics seems to exist between gonadotropins and Amh, as evidenced by the direct inhibitory effect of Fsh on *amh* expression in fish testes [42,43,44,45,46], in contrast to the stimulatory effect reported in mammals [8,47]. So far, no consistent pattern for androgenic action on *amh* expression exists in teleosts, likely reflecting species-specific regulatory activity [23]. These findings show the need for further research to clarify how Amh integrates into the complex regulatory network that maintains the balance among gonadal growth, gametogenesis, renewal of germ cells, and differentiation into mature gametes.

In the European sea bass (*Dicentrarchus labrax*), a well-established model for marine fish endocrinology [48,49,50], *amh* and *amhr2* were previously isolated [18,51] and functionally characterized by using homologous recombinant Amh produced in Chinese hamster ovary (CHO) cells [51] or *Pichia pastoris* [52]. Gene expression has been studied across various tissues and in adult gonads throughout the annual reproductive cycle, and protein localization in the ovary and testis has been achieved using specific antibodies [51,52]. Notably, the synergistic effect of Amh on Fsh-induced steroidogenesis in pre-vitellogenic ovaries of European sea bass was recently demonstrated for the first time in any vertebrate, supporting a role for Amh in ovarian estradiol production [52]. Building on this finding, the present study explores the regulatory role of Amh in male European sea bass reproductive processes, with a focus on steroidogenesis. Leveraging the availability of homologous recombinant Amh, currently possible in only a few fish species, *in vitro* experiments assess its direct effects on testis physiology and compare them with those previously found in females to confirm its steroidogenic activity. Complementarily, an *in vivo* approach using intramuscular injection of an *amh* expression plasmid enables exploration of Amh effects on steroid production within the context of an active BPG axis. Finally, to elucidate the mechanisms underlying Amh-induced steroidogenesis, the involvement of canonical SMAD and cyclic adenosine monophosphate (cAMP) pathways was examined via an Amhr2-mediated transactivation assay in cell line from African green monkey kidney fibroblasts (COS-7 cells).

## 2. Results

### 2.1. Localization of Endogenous Amh Type II Receptor, Amhr2, in Pre-Meiotic Testis of Adult Sea Bass

**Figure 1:**
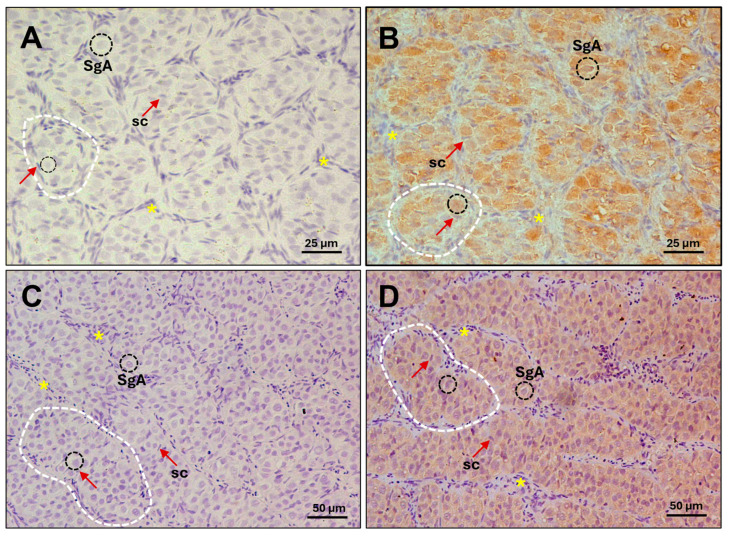
Immunohistochemical localization of sea bass Amhr2 in pre-meiotic testis from adult specimens. Photomicrographs from (**A**,**B**) August and (**C**,**D**) October showing immature stage. Representative cell types and structures are indicated: type A spermatogonia (SgA, black dashed circles); Sertoli cell (sc, nucleus marked by red arrows; these supporting cells can be identified by distinct unstained white lines encircling germ cells); lobules (dashed white lines); interstitial cells (asterisks). (**A**,**C**) Control sections without primary antibody; (**B**,**D**) Amhr2 staining signal (brown colour) in sections incubated with 10 µg/mL of anti-Amhr2 primary antibody. Bars = 25 µm (**A**,**B**), 50 µm (**C**,**D**).

Adult European sea bass males in the pre-meiotic stage (August–October) were used to immunolocalize the endogenous Amhr2. At this developmental stage, the testis is composed of type A spermatogonia, each individually surrounded by Sertoli cells. The type A spermatogonia are organized into well-defined lobules, delineated by interstitial cells. The sea-bass-specific anti-Amhr2 antibody clearly detected the receptor within type A spermatogonia at both time points, both before the onset of spermatogenesis (August, Figure 1A,B) and at the beginning of spermatogenesis before meiosis starts (October, Figure 1C,D) to initiate the new reproductive cycle. These findings confirm the presence and localization of the specific type II receptor Amhr2 in pre-meiotic testes, thereby ensuring the appropriateness of these animals for the subsequent functional assays involving Amh.

### 2.2. Effect of Sea Bass Amh on Steroidogenesis in Fish Testis

**Figure 2:**
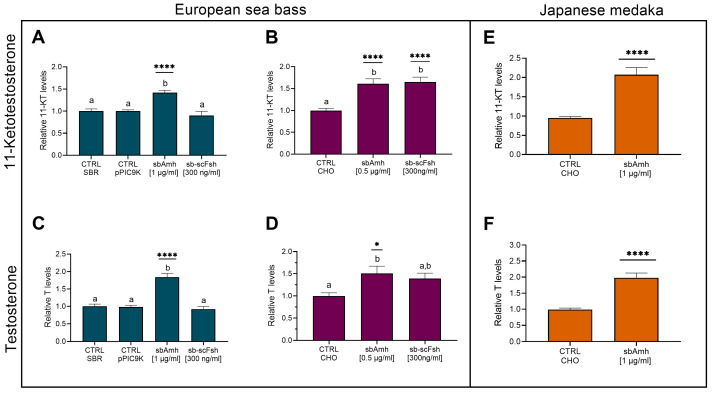
Levels of sexual steroids in culture medium of testis. (**A**,**B**,**E**) 11-ketotestosterone and (**C**,**D**,**F**) testosterone in (**A**–**D**) pre-meiotic testis from adult European sea bass and (**E**,**F**) testis from adult Japanese medaka, stimulated with recombinant sbAmh produced in (**A**,**C**; N = 5) *P. pastoris* or in (**B**,**D**, N = 6; **E**,**F**, N = 43) CHO cells. Steroid contents quantified as ng/mg (**A**–**D**) or as ng/mL of culture medium (**E**,**F**) and expressed as fold change in control treatment (CTRL SBR or CTRL CHO), which was set as 1. Data reported as mean ± SEM. Letters represent significant differences (*p* < 0.05) among treatments; asterisks indicate significant differences (* *p* < 0.05; ** *p* < 0.01; *** *p* < 0.005; **** *p* < 0.001) in steroid levels compared to control treatment. CTRL SBR: Control Sea Bass Ringer; CTRL pPIC9K: *P. pastoris* sham culture medium; CTRL CHO: CHO cells sham culture medium.

To investigate the effect of Amh on steroidogenesis, pre-meiotic testes from adult European sea bass (gonadodomatic index, GSI = 0.104 ± 0.0095 g [mean ± SEM]) were cultured *in vitro* and treated with recombinant sea bass Amh (sbAmh) or sea bass single-chain Fsh (sb-scFsh). Steroid levels (11-Ketotestosterone, 11-KT and Testosterone, T) in the culture medium were measured by using specific enzyme immunoassays (EIA). Both recombinant sbAmh types, the one produced in yeast *P. pastoris* and the one from CHO cells, induced a significant increase of 11-KT (Figure 2A,B) and T (Figure 2C,D) levels in the culture medium compared to testis incubated with the control media (SBR, pPIC9K, CHO). Testosterone production was not affected by sb-scFsh (Figure 2C,D) treatments, which instead induced a significant increase in 11-KT content (Figure 2B). To evaluate if this steroidogenic effect of Amh was unique to sea bass testis, incubations were performed as described above using testis from adult reproductive competent Japanese medaka, the model species phylogenetically closest to the sea bass. These *in vitro* experiments showed a marked ability of sbAmh to significantly increase both 11-KT and T levels (Figure 2E,F) in the culture medium compared to control treatment.

**Figure 3:**
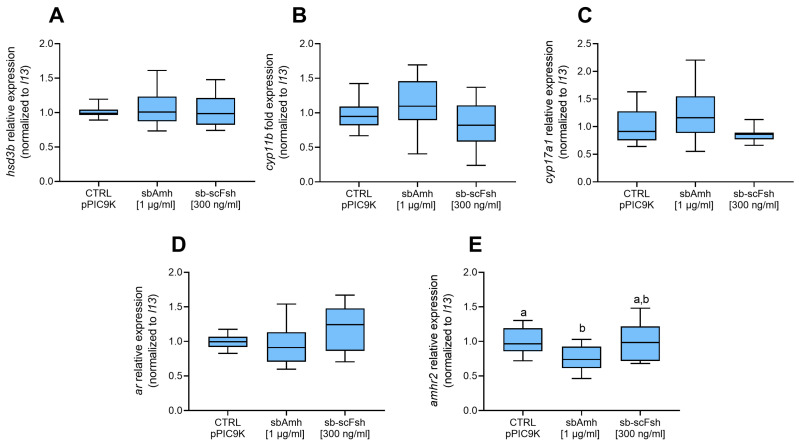
Relative gene expression of (**A**) *hsd3b*, (**B**) *cyp11b*, (**C**) *cyp17a1*, (**D**) *ar*, and (**E**) *amhr2* in pre-meiotic sea bass testis explants (N = 5). Data reported as mean ± SEM, normalized to housekeeping and expressed as fold change in control treatment (CTRL pPIC9K), which was set as 1. Letters represent statistical significance (*p* < 0.05) among different treatments. CTRL pPIC9K: *P. pastoris* sham culture medium.

The tissue cultured *in vitro* was used to investigate the possible effect of sbAmh on the expression of the genes coding for steroidogenic enzymes, androgen receptor, and AmhR2. No significant differences in *hsd3b* expression were observed when testis was treated with recombinant sbAmh produced in *P. pastoris* or with recombinant sb-scFsh compared to the control treatment (Figure 3A). Slight fluctuations could be appreciated in the expression of *cyp11b*, *cyp17a1*, and *ar*, but none were statistically significant (Figure 3B–D). The only notable effect induced by sbAmh was a significant downregulation of *amhr2* expression compared to both the control and sb-scFsh treatments, which showed similar expression levels (Figure 3E). Consistent results were also obtained when the testes were treated with recombinant sbAmh produced in CHO cells, compared to the CHO sham medium treatment.

### 2.3. In Vivo Effect of sbAmh

**Figure 4:**
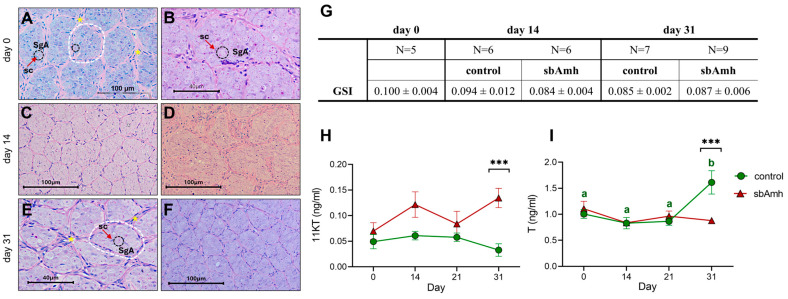
Effect of sea bass Amh expression plasmid injected intramuscularly in adult male specimens. Representative histological photomicrographs of testis at (**A**,**B**) day 0 (not injected), (**C**,**D**) day 14, and (**E**,**F**) day 31 in (**A**,**C**,**E**) control and (**B**,**D**,**F**) sbAmh groups. SgA, type A spermatogonia (black dashed circles); sc, Sertoli cell (nucleus marked by red arrows; these supporting cells can be identified by distinct unstained white lines encircling germ cells); lobules (dashed white lines); interstitial cells (asterisks). (**G**) GSI mean ± SEM for each group and time point. (**H**) 11-ketotestosterone (11-KT) and (**I**) testosterone (T) plasma levels throughout experiment. Steroid contents quantified as ng/mL plasma. Data reported as mean ± SEM. Letters represent statistical significance (Kruskal–Wallis + Dunn’s) (*p* < 0.05) between time points within experimental group (control or sbAmh); asterisks indicate statistical difference (Mann–Whitney) (* *p* < 0.05; ** *p* < 0.01; *** *p* < 0.005; **** *p* < 0.001) between groups at each time point.

To investigate the steroidogenic activity of Amh *in vivo*, adult sea bass males were injected with the sea bass Amh expression plasmid pcDNA3-sbAmh (sbAmh group) or the empty pcDNA3 vector (control group). Assessment of testis maturity stage at the beginning (day 0), middle (day 14), and end of the trial (day 31) showed the same histological morphology throughout the experiment and in both experimental groups, demonstrating that the treatment did not affect gonad development (Figure 4A–F). In fact, the testes were in the pre-meiotic stage, characterized by type A spermatogonia surrounded by Sertoli cells, and organized into lobules, which are delineated by interstitial cells. Similarly, GSI did not vary during the trial, showing no significant differences between groups or treatments (Figure 4G).

The plasma steroid levels of both 11-KT and T, evaluated in the same animals from the beginning to the end of the trial (control group, N = 7; sbAmh group, N = 9), were affected by Amh but in an opposite way. In particular, the control group maintained consistent low levels of 11-KT during the entire duration of the experiment, while in the other group sbAmh induced fluctuations of the hormone up to a significant increase at the end of the trial (day 31) compared to the control group (Figure 4H). The opposite trend was recorded for T, which exhibited the same levels in the two groups until day 21 and then, at the end of the trial, became significantly higher in the control group than in the sbAmh group (Figure 4I). Unlike the effect on steroid production, sbAmh did not induce any significant changes in the expression of genes coding for steroidogenic enzymes, exhibiting results consistent with those obtained by tissue culture *in vitro*.

### 2.4. Signalling Pathways of sbAmh

#### 2.4.1. sbAmh Signalling Through the SMAD and cAMP Pathways

**Figure 5:**
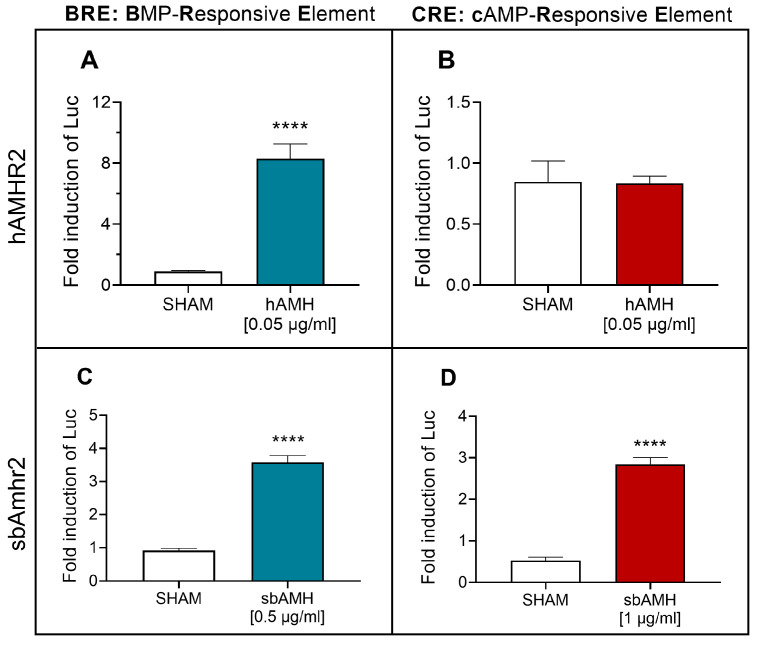
Human AMH (hAMH) and recombinant sea bass Amh (sbAmh) actions on SMAD (BRE-Luc) and cAMP (CRE-Luc) signalling pathways tested in COS-7 cells. hAMH induction of (**A**) BRE-Luc and (**B**) CRE-Luc reporter activities through hAMHR2. sbAmh induction of (**C**) BRE-Luc and (**D**) CRE-Luc reporter activities through sbAmhr2. Firefly luciferase activities measured as relative light units (RLU) and expressed as fold inductions in respect to control 1% FBS DMEM. Data reported as mean ± SEM. Asterisks represent statistical significance (* *p* < 0.05; ** *p* < 0.01; *** *p* < 0.005; **** *p* < 0.001) of each treatment compared to sham. SHAM: (**A**,**B**) 1% FBS DMEM with hAMH resuspension buffer (4 mM HCl, 0.1% BSA in PBS); (**C**,**D**) CHO cells sham culture medium.

Transactivation assays of the specific Amh type II receptor (sbAmhr2) were performed in COS-7 cells to delve deeper into the steroidogenic activity of sbAmh by assessing activation of the cAMP pathway in comparison with its ability to signal through the canonical SMAD pathway. The activation of these intracellular pathways was detected by means of BRE-Luc or CRE-Luc reporter constructs for the SMAD and cAMP pathways, respectively. These same trials were performed with human Amh (hAMH) signalling through its specific type II receptor (hAMHR2). hAMH significantly activated the SMAD pathway (Figure 5A) compared to the control, whilst no action was detected on the cAMP pathway (Figure 5B). These effects were confirmed by testing different doses. The functionality of hAMH on its canonical pathway was demonstrated even at lower doses (Figure S1A), while no dose triggered the cAMP pathway (Figure S1B). SbAmh was able to significantly activate the canonical SMAD pathway through its cognate sbAmhr2 (Figure 5C), and the same effect was detected for the cAMP pathway (Figure 5D). Other doses were also tested, and sbAmh significantly activated the cAMP pathway at lower doses (0.5 µg/mL), although to a lesser extent (Figure S1C).

#### 2.4.2. Crosstalk Between Signalling Pathways

**Figure 6:**
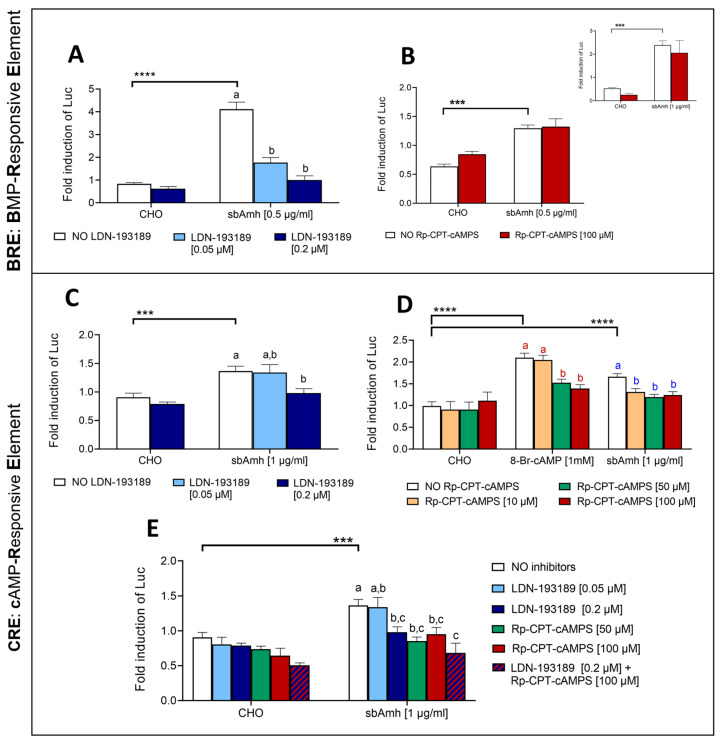
The effect of specific inhibitors on the SMAD (BRE-Luc) and cAMP (CRE-Luc) signalling pathways stimulated by sbAmh in COS-7 cells transiently transfected with sbAmhr2. (**A**,**B**) The SMAD pathway. (**C**–**E**) The cAMP pathway. The effect of (**A**,**C**) the specific SMAD pathway inhibitor LDN-193189 hydrochloride and (**B**,**D**) the specific cAMP inhibitor Rp-8-CPT-cAMPS on (**A**,**B**) BRE-Luc and (**C**,**D**) CRE-Luc reporter activities. (**E**) The effect of the combined action of LDN-193189 hydrochloride and Rp8-CPT-cAMPS on CRE-Luc reporter activity. The incubations in (**A**–**C**) were performed for 24 h, and those in (**D**,**E**) were performed for 4 h. The inlet in B shows the same incubation for 16 h. Firefly luciferase activities were measured as RLU and expressed as fold inductions of luciferase activity respect to the control 1% FBS DMEM. The data are reported as means ± SEM. The letters indicate statistical differences (*p* < 0.05) among inhibitory treatments within the same stimulatory group (ANOVA + Tukey’s). Different colors of letters correspond to different stimulatory groups. The asterisks represent statistical significance (Student *t*-test) (* *p* < 0.05; ** *p* < 0.01; *** *p* < 0.005; **** *p* < 0.001) between the stimulatory treatment and CHO in the absence of inhibitors. CHO: CHO cells sham culture medium.

The addition of pathway-specific inhibitors allowed us to investigate possible crosstalk between the SMAD and cAMP signalling pathways. SbAmh significantly activated the SMAD pathway after 24 h of treatment, and both tested concentrations of LDN-193189 hydrochloride specific inhibitor were able to significantly counteract this action, bringing luciferase values close to their basal levels when the highest concentration of 0.2 µM was used (Figure 6A). In a similar way, sbAmh induced a significant increase in CRE-driven luciferase activity after 4 h of stimulation, and the Rp-8-CPT-cAMPS inhibitor was able to hinder this effect when used at 50 µM and 100 µM (Figure 6D). The functionality of Rp-8-CPT-cAMPS was confirmed by its ability to inhibit the stimulatory effect of the cAMP analogue 8-Br-cAMP on CRE-driven luciferase activity (Figure 6D), although a shorter incubation period was necessary to ensure these results (Figure S2).

The significant activation of the BRE-Luc promoter induced by sbAmh was not reduced by addition of Rp-8-CPT-cAMPS (Figure 6B), demonstrating that this inhibitor has no effect on the SMAD pathway. Since Amh requires at least a 20 h treatment to have a measurable effect on BRE-Luc but Rp-8-CPT-cAMPS loses its functionality after such a long incubation, the experiment was repeated, reducing the treatment to 16 h to ensure that the lack of effect of Rp-8-CPT-cAMPS on BRE was not due to the long incubation time. As shown in the inlet of Figure 5B, the results with 24 h of incubation were confirmed after 16 h: sbAmh induced its canonical pathway but Rp-8-CPT-cAMPS could not inhibit this effect.

In the same way, the action of LDN-193189 hydrochloride on the cAMP pathway was tested for 24 h, and the highest dose was able to significantly reduce the stimulatory action of sbAmh (Figure 6C), indicating possible crosstalk between the two pathways. When the highest concentration of LDN-193189 and Rp-8-CPT-cAMPS were added together to the treatment, their inhibitory effect on sbAmh-stimulated CRE-Luc activation was more evident, though not significantly different from the actions of both inhibitors separately (Figure 6E). These results indicate that both LDN-193189 and Rp-8-CPT-cAMPS are capable of counteracting cAMP activation, suggesting a potential synergistic effect of the two inhibitors.

### 2.5. Involvement of cAMP Pathway in sbAmh-Stimulated Steroid Production in Testis

**Figure 7:**
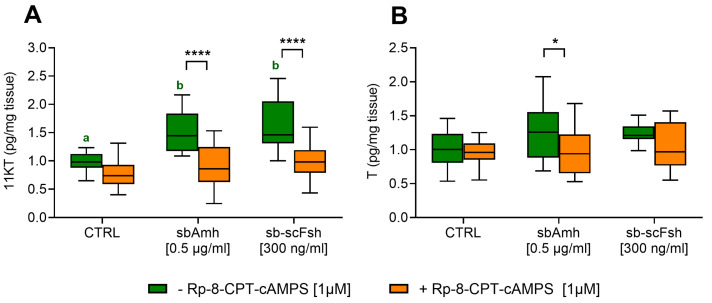
The levels of (**A**) 11-ketotestosterone and (**B**) testosterone in the culture medium of pre-meiotic testis from adult European sea bass (N = 5). Steroid contents were quantified as pg/mg tissue and expressed as fold change with respect to the control treatment (CTRL: CHO sham culture medium without inhibitor), which was set as 1. Data are reported as mean ± SEM. The letters represent statistical significance (*p* < 0.05) among stimulatory treatments (ANOVA + Tukey’s). The asterisks indicate statistical difference (ANOVA + Sidak’s) (* *p* < 0.05; ** *p* < 0.01; *** *p* < 0.005; **** *p* < 0.001) with or without an inhibitor within stimulatory treatments.

The cAMP pathway inhibitor Rp-8-CPT-cAMPS was employed in testis culture to confirm the involvement of this signalling pathway in sbAmh-induced steroid production. Additionally, sb-scFsh was included as positive control, as it is known to stimulate steroidogenesis primarily through cAMP-dependent signalling. 11-KT levels significantly increased when testis explants were treated with sbAmh or sb-scFsh, and in both cases Rp-8-CPT-cAMPS totally abolished this effect (Figure 7A). The production of T, a precursor of 11-KT, was slightly stimulated by sbAmh and inhibited by the addition of Rp-8-CPT-cAMPs (Figure 7B).

## 3. Discussion

The multidisciplinary approach employed in the present study revealed that the Anti-Müllerian hormone exerts a steroidogenic function in two distinct teleost species, European sea bass and Japanese medaka. This represents a novel finding in teleosts, as no previous studies have reported a similar role for Amh in fish. Initial evidence for this action came from a previous work conducted by our group, which revealed that Amh has an additive effect on Fsh-stimulated steroidogenesis in female European sea bass, increasing *cyp19a1a* expression and estrogen production in adult pre-vitellogenic ovaries cultured *in vitro* [52]. The current study corroborates these previous findings, reporting a significant Amh-induced stimulation of androgen production. The use of biologically active recombinant sea bass Amh produced in CHO cells [51] and *P. pastoris* yeast [52], previously developed by our group, enabled *in vitro* testing of both hormone forms, thereby confirming their stimulatory effect on steroid production.

Steroidogenesis is a highly regulated and complex process involving multiple components, hormones such as gonadotropins, growth factors, enzymes, and receptors, that cooperate to ensure efficient signalling. In mammals, FSH and LH regulate gonadal development via distinct receptors on specific cell types: FSH acts on Sertoli cells to support germ cell development, while LH stimulates steroid production in Leydig cells [53]. In teleosts, however, this division of roles is clearly less defined. In some species Fsh not only targets Sertoli cells via the Fsh receptor (Fshr), but also directly stimulates steroidogenesis by acting on Fshr present in Leydig cells [54,55]. This dual action, along with possible receptor cross-activation in certain species [56,57], adds further complexity to the regulatory network. In European sea bass, as in many teleost species, Fsh is present in early stages of gametogenesis, whereas Lh is involved in the final phases [58,59,60].

In the present study, pre-meiotic testes were cultured, using Fsh as a reference steroidogenic stimulus. Although previous studies in European sea bass demonstrated that Fsh stimulates androgen production, both *in vitro* and *in vivo* [46,61,62], this effect has only been observed after the onset of spermatogenesis. Indeed, no increase in androgen levels was detected in pre-meiotic testis (September) treated with purified native sea bass Fsh [62], as also occurred in some of the trials in this study.

Unlike Fsh, recombinant Amh elicited a clear steroidogenic response in the pre-meiotic testis, significantly increasing androgens (11-KT and T) in both *in vitro* trials. This is the first report of such an effect for Amh in teleosts, indicating that pre-meiotic testes possess the molecular components required for Amh signalling. In fact, Rocha et al., (2016) [51] reported high expression levels of *amhr2* in adult immature testes.

Consistently, this study confirmed the presence of Amhr2 in type A spermatogonia through immunolocalization, confirming that Amh action could be ensured by the presence of its specific type II receptor during early testicular development. This aligns with previous findings in females, where Amhr2 was detected in oocytes of pre-vitellogenic and vitellogenic follicles [52], The identification of Amhr2 in type A spermatogonia supports a paracrine role for Amh, produced by adjacent Sertoli cells. Moreover, the strong immunoreactivity observed may reflect elevated Amhr2 protein synthesis at this developmental stage.

The limited availability of homologous recombinant Amh (rAmh) in teleosts has constrained progress in functional studies on its mechanisms of action and regulatory role in gonadal physiology, resulting in scarce data comparable to the present findings. To date, rAmh has been produced only in Japanese eel [14], zebrafish [43], black porgy (*Acanthopagrus schlegelii*) [38] and spotted steed (*Hemibarbus maculatus*) [63]. Of these, only the zebrafish study directly addressed the involvement of Amh in male steroidogenesis. In particular, co-treatment of adult zebrafish testis with homologous rAmh and recombinant Fsh revealed that Amh suppresses Fsh-induced 11-KT production, demonstrating an inhibitory effect on steroidogenesis [43]. In contrast, studies in Japanese eel and black porgy did not examine steroidogenesis, but showed that rAmh inhibits proliferation of type A spermatogonia [14,38].

In mammals, the inhibitory role of AMH on steroidogenesis is well established. In the ovary, AMH directly regulates the steroidogenic enzymes, reducing estrogen synthesis and follicular sensitivity to FSH [64,65,66]. In the testis, AMH antagonizes LH action in the control of androgen production, hindering Leydig cell proliferation and differentiation [67].

By contrast, our findings in European sea bass reveal a stimulatory effect of Amh on steroidogenesis, prompting further investigation in a phylogenetically close teleost model, the Japanese medaka, where the role of Amh in steroidogenesis remains unknown [23]. As no homologous rAmh is available for medaka, we used sbAmh for *in vitro* testis culture experiments. Remarkably, sbAmh elicited a potent steroidogenic response in medaka testes, exceeding that observed in sea bass and significantly increasing androgen production. The effectiveness of sbAmh in medaka is likely based on the conserved protein sequence between the two species: Amh from medaka and sea bass shares 62.16% protein sequence identity in the C-terminal bioactive domain. This region contains the conserved cysteine residues essential for dimerization and biological activity of TGF-β family members [68], supporting functional cross-species activity.

Despite the clear increase in androgen levels induced by sbAmh, no significant changes were observed in the expression of key steroidogenic genes. Synthesis of steroids involves a complex cascade of oxidative enzymes that convert cholesterol into active hormones; however, the regulatory mechanisms governing these enzymes are not clearly understood [69]. The regulation of steroid hormone biosynthesis varies depending on whether the stimulus is acute or chronic. Chronic stimuli can induce transcriptional regulation of steroidogenic genes, while acute stimuli influence mainly cholesterol transport into the mitochondria [70,71,72,73]. Given that *in vitro* treatments with sbAmh may be considered an acute stimulus, gene expression variation may not be detectable.

Changes in gene expression induced by Amh have been documented in only a few species. In zebrafish testis cultures, rAmh suppressed the Fsh-induced upregulation of the steroidogenic genes *cyp17a1* and *star* [43]. In spotted steed, rAmh treatment reduced both E2 levels and *cyp19a1* expression in oocytes [63]. Conversely, in European sea bass ovary, *cyp19a1* expression increased following rAmh treatment [52]. These findings suggest that Amh may exert species- and sex-specific regulatory effects on steroidogenesis.

Moreover, the androgen increase observed may involve non-transcriptional mechanisms. Post-translational modifications could enhance enzyme activity, increase substrate availability, or alter the enzyme kinetics, thereby boosting steroid output without requiring changes in gene expression [74,75]. Further investigation is needed to elucidate the underlying regulatory mechanisms. Interestingly, Amh treatment induced a significant downregulation of *amhr2* expression compared to the control. This suggests the activation of a negative feedback mechanism, possibly triggered by a rapid hormone increase, aimed at reducing tissue responsiveness to Amh. Ligand-induced receptor downregulation is a well-known regulatory mechanism and has been described for various hormones, including estradiol [76], gonadotropins [77], and Amh itself [78].

To investigate the *in vivo* steroidogenic activity of Amh, adult male sea bass with pre-meiotic testes were injected with an sbAmh expression plasmid. The results were consistent with the *in vitro* testis culture, confirming a local steroidogenic action for Amh. Plasma 11-KT levels gradually increased, reaching statistical significance by day 31. This delayed response likely reflects the time required for plasmid expression and downstream steroidogenic effects, consistent with previous reports showing peak levels of the encoded protein in circulation around 14 days post-injection [46]; in this case, the delay may be longer since 11-KT is downstream of Amh action. In the control group, 11-KT plasma levels remained stable, while testosterone increased by the end of the trial, consistent with the onset of the reproductive season in September. These trends agree with the natural steroid profiles in adult male sea bass, where T started to increase between August and September [79,80]. Notably, the Amh-treated group did not show the testosterone peak seen in controls at day 31, suggesting rapid conversion of testosterone to 11-KT under Amh stimulation.

These findings support a gonad-localized role of Amh in steroidogenesis, independent of central regulation via the BPG axis and likely mediated by testicular factors responsive to Amh signalling. However, the exact interplay between gonadotropins, androgens, and Amh in sea bass remains unclear, leaving open the possibility of additional regulatory layers at the level of the BPG axis. Reviewing existing studies on Amh in both mutant [35,37,81] and wild-type teleosts [44] reveals that Amh does not exert a single, conserved function across species. Rather, its role appears to be species-specific and strongly influenced by the reproductive stage examined. Nonetheless, a general consensus suggests that Amh acts as a regulator of gametogenesis without broadly inhibiting spermatogenesis or compromising fertility [82]. It contributes to gonadal homeostasis throughout the reproductive lifespan, acting as an inhibitor of the proliferation of mitotically self-renewing germ cells and as a permissive factor for the progression of spermatogonial differentiation toward meiosis, thus maintaining balanced germ cell progression [35,36,37].

**Figure 8:**
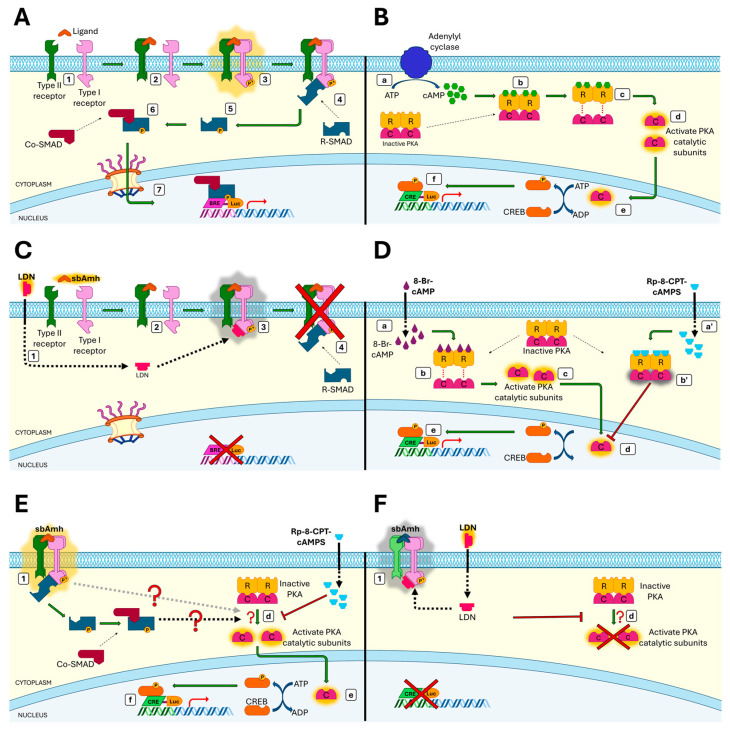
The role of sea bass Amh in canonical TGF-β/Smad pathway and steroidogenic PKA-cAMP pathway. (**A**) The Smad signalling pathway consists of extracellular ligands, cell surface type I, type II serine-threonine kinase receptors, and intracellular Smad proteins (1). Dimers of ligand (sbAmh) bind to type II receptor dimers (sbAmhr2) (2) and recruit type I receptor dimers (ALK2/3), which are phosphorylated, leading to an activated tetrameric type II/type I receptor complex (3). The phosphorylated type I receptor recruits cytosolic R-Smad proteins (SMAD 1/5/8) (4) and phosphorylates the protein, inducing a conformational change (5) which leads to the activation of R-Smad to form hetero-oligomeric complexes with Co-Smad (SMAD4) (6). Finally, the Smad complexes are translocated to the nucleus where they bind to the promoter (BRE) and regulate the expression of target genes (7). (**B**) cAMP/PKA signalling is the main steroidogenic pathway triggered by the increase in intracellular cAMP concentration. The activated adenylyl cyclase enzyme catalyzes the conversion of ATP into cyclic adenosine monophosphate (cAMP) (a), and the rise in concentration of the second messenger cAMP causes binding of cAMP to the cyclic-AMP-dependent protein kinase (PKA) (b). Inactive PKA is a cytosolic tetrameric holoenzyme composed of two regulatory subunits (PKA-R) associated with two catalytic subunits (PKA-C); binding of cAMP to the PKA-R causes the dissociation of the complex (c), allowing the free catalytic subunits to be active as serine/threonine kinases in the cytoplasm (d). The active holoenzyme enters the nucleus (e) where it induces the phosphorylation of the cAMP response element-binding protein (CREB). Finally, the CREB transcription factor binds to the promoter (CRE) and regulates the expression of target genes (f). (**C**) LDN193189 hydrochloride (ALK2/3 inhibitor), administrated prior the sbAmh, crosses the plasma membrane and binds to the type I receptor (1). When the sbAmh is added, it first recruits the sbAmhr2 dimer (2), then the type I dimer (3), but the presence of LDN prevents the activation of the complex (3) and the subsequent downstream cascade of the Smad pathway, hindering BRE-driven gene expression (4). (**D**) 8-Br-cAMP is a membrane-permeant activator of PKA that, once it has entered the cells (a), binds to the PKA regulatory subunits (b), mimicking cAMP and thus inducing the activation of PKA-C (c). As with the increase in intracellular cAMP, the final result is the phosphorylation of CREB in the nucleus (d) and the regulation of CRE-driven gene expression (e). When Rp-8-CPT-cAMP is present, this mechanism is blocked: this compound is a potent membrane-permeant inhibitor of PKA (a′) which binds to PKA-R, avoiding the dissociation of catalytic subunits (b′) and thus CREB phosphorylation. (**E**) When the sbAmh/sbAmhr2/type I tetrameric complex is activated (1), the dissociation of PKA catalytic subunits occurs (d), leading to CREB phosphorylation in the nucleus (e) and CRE-Luc activation (f). Rp-8-CPT-cAMP prevents PKA activation, blocking the effect of sbAmh on the cAMP/PKA pathway. sbAmh-induced CRE-Luc activation is mediated by activate PKA, but it is still unknown if PKA activation is Smad-dependent (black dashed arrow) or not (grey dashed arrow). (**F**) LDN193189 hydrochloride prevents the activation of the sbAmh/sbAmhr2/type I tetrameric complex (1) and, parallel to it, the activation of PKA does not occur (d), demonstrating that the effect of sbAmh on the steroidogenic cAMP/PKA pathway is mediated by the type I receptor belonging to the TGF-β superfamily.

The intracellular signalling pathways activated by sbAmh through its cognate receptor Amhr2 were investigated using transactivation assays in COS-7 cells. According to the literature, COS-7 cells, which lack endogenous Amhr2 expression [83,84] but express the three type-1 receptors Acvr1 (Alk2), Bmpr-1a (Alk3), and Bmpr1b (Alk6) [51], known to be recruited by Amhr2 in mammals [85], represent a suitable model for studying Smad pathway activation. Previous studies demonstrated that recombinant sbAmh induces dose-dependent activation of the BRE-Luc reporter via Amhr2 [51,52]. Our results confirmed these findings and showed that this activation was significantly inhibited by LDN193189 hydrochloride, a potent inhibitor of the type I receptors Alk2 and Alk3, showing high selectivity over other type I receptors of the TGF-β family [86,87]. This inhibition blocks activation of the heterodimeric receptor complex upon sbAmh binding (Figure 8C). Together, these results provide the first evidence in teleosts that mature sbAmh signals through its specific type II receptor Amhr2 by recruiting Alk2 or Alk3 as type I receptors, mirroring the conserved mechanism described in mammals.

We also explored whether sbAmh activates non-canonical intracellular pathways related to its steroidogenic effects, focusing on the cAMP/PKA pathway, which is known to mediate gonadotropin-induced steroidogenesis [88,89]. Gonadotropin signalling induces an increase in intracellular cAMP, which in turn activates protein kinase A (PKA), leading to the phosphorylation of downstream substrates (Figure 8B) [90,91]. In Leydig cells, PKA regulates steroidogenesis via phosphorylation of key enzymes and transcription factors [73]. Using CRE-Luc reporter assays in COS-7 cells, we found that sbAmh stimulated CRE-driven luciferase activity in an Amhr2-dependent and dose-dependent manner, similar to the effect of 8-Br-cAMP, a cAMP analogue used as the positive control. In contrast, human AMH activated the Smad pathway via hAMHR2 but failed to induce CRE-Luc activity, in line with the lack of evidence of mammalian AMH inducing steroid synthesis.

To confirm the involvement of the non-canonical cAMP/PKA pathway, we used Rp-8-CPT-cAMPs, a competitive antagonist of cAMP that blocks protein kinase A (PKA) activation [92,93], since it competes with cAMP for binding to the PKA regulatory subunits [94] and prevents the activation of PKA catalytic subunits and downstream signalling (Figure 8D). Co-treatment with Rp-8-CPT-cAMPs abolished CRE-Luc activation induced by both 8-Br-cAMP and sbAmh (Figure 8E), confirming PKA dependency. In testis explants, Rp-8-CPT-cAMPs similarly blocked the 11-KT synthesis induced by both recombinant sb-scFsh and sbAmh. These results strongly support that active PKA is required for sbAmh-mediated steroidogenesis in sea bass.

The mechanism by which Amh activates both Smad and cAMP/PKA pathways remains unclear. To investigate potential crosstalk, we applied specific inhibitors of each pathway to test for effects on the alternate pathway. This approach is supported by studies in mammals indicating interaction between these pathways [95]. In the canonical Smad pathway, TGFβ ligands activate their receptors leading to C-terminal phosphorylation of R-Smads (Figure 8A). Non-canonical Smad signalling also exists, wherein activated receptors recruit intracellular serine/threonine kinases that phosphorylate R-Smads in the linker region [96].

Although typically activated by cAMP, PKA can also be activated via cAMP-independent mechanisms. In murine mesangial cells, TGFβ1 increases PKA activity and cAMP response element-binding protein (CREB) phosphorylation without altering cAMP levels [97] through a Smad-dependent process involving Smad4 [98]. Specifically, Smad3, activated by TGFβ, recruits Smad4, which directly interacts with the regulatory subunit of PKA (PKA-R), forming a trimeric complex that activates PKA independently of cAMP [99], a process that can be blocked by the PKA inhibitor H89 [98]. This is consistent with our finding that sbAmh-induced CRE-Luc activity is suppressed by Rp-8-CPT-cAMPs, while Smad-dependent BRE-Luc activation remains unaffected by the same inhibitor, indicating that PKA does not modulate canonical sbAmh signalling.

This is further supported by Zhang et al. (2004), who showed that PKA inhibition does not affect TGFβ receptor kinase activity, receptor–Smad complex formation, or Smad activation [98]. Conversely, inhibition with LDN193189 inhibitor, which targets Alk2/3, blocked sbAmh-induced CRE-Luc activity (Figure 8F), suggesting that Alk2/Alk3 type I receptors mediate activation of the cAMP/PKA pathway in sea bass. Collectively, these results provide evidence of functional crosstalk between Smad and cAMP/PKA signalling in sea bass.

Finally, the interaction of AMH with the cAMP/PKA signalling route has been observed in human granulosa cells, where AMH modulates Stem Cell Factor (SCF) expression by promoting CREB phosphorylation via the cAMP/PKA pathway, an effect that could be partially blocked by the PKA inhibitor H89 [100,101].

## 4. Materials and Methods

### 4.1. Animals

Adult male European sea bass (*Dicentrarchus labrax*) were individually tagged (PIT tags; Avid identification System, Inc. Norco, California, USA) and reared at the facilities of the Instituto de Acuicultura Torre de la Sal (IATS, Castellón, Spain, 40° N) under natural photoperiod and temperature conditions. The fish were anesthetized with an overdose of ethyl 3-aminobenzoate methanesulfonate salt (MS-222; 300–400 mg/L; Sigma-Aldrich^®^, St. Louis, MO, USA) and euthanized by decapitation according to Spanish Royal Decree (53/2013) and European legislation (2010/63/EU) for the protection of animals used for scientific purposes. The protocol was approved by the IATS Ethics Committee (Register Number 09-0201) under the supervision of the Secretary of State for Research, Development and Innovation of the Spanish Government. For each sacrificed animal, developmental stage of gonad was determined by histological analysis, following previously established criteria [102]. Medaka were kept and sampled in accordance with the applicable EU and national German legislation governing animal experimentation. In particular, all experimental protocols were approved through an authorization (55.2532-2-215) of the Veterinary Office of the District Government of Lower Franconia, Germany, in accordance with the German Animal Protection Law (TierSchG) and in accordance with ARRIVE guidelines.

### 4.2. Hormones and Reagents

Human AMH (hAMH) was obtained from R&D Systems, Inc. (Minneapolis, MN, USA). Recombinant sea bass Fsh and Amh were produced in-house. Sea bass single-chain Fsh (sb-scFsh) was produced in CHO cells as described previously [61]. Recombinant sea bass Amh (sbAmh) was obtained from a *Pichia pastoris* clone expressing the pPIC9K-sbAmh plasmid [52] or produced in CHO cells from a stable clone generated in a previous study [51]. When using CHO-derived hormones, the control treatments consisted of culture medium obtained from CHO cells expressing the empty pcDNA3 vector. Culture medium obtained from *P. pastoris* expressing the empty pPIC9K vector was used as the control for Amh produced in yeast.

The following signalling pathway inhibitors were used in combination with hormones: LDN-193189 hydrochloride (Sigma-Aldrich^®^, St. Louis, MO, USA), a highly selective antagonist of BMP receptor isotypes ALK2 and ALK3 was used to inhibit the SMAD pathway and Rp-8-CPT-cAMPS (BIOLOG Life Science Institute, Bremen, Germany), a cAMP-dependent protein kinase type I and type II inhibitor, was used for the inhibition of the cAMP pathway. The 8-Br-cAMP (BIOLOG Life Science Institute, Bremen, Germany), a membrane-permeant activator of cAMP-dependent protein kinase type I and type II, analogue of the natural cAMP, was used as a positive control for cAMP pathway activation in cell culture experiments.

### 4.3. Immunohistochemistry of Endogenous Amhr2 in Adult Sea Bass Testis

Premeiotic testis from adult sea bass were fixed overnight at 4 °C in 4% paraformaldehyde (PFA) in PBS, then dehydrated and embedded in paraffin. Sections of approximately 5 µm thickness were deparaffinized in xylene, rehydrated in decreasing concentrations of ethanol, and washed twice with double-distilled water. Slides underwent heat-induced antigen retrieval by Tris-EDTA Buffer (10 mM Tris Base, 1 mM EDTA, and 0.05% Tween 20, pH 9.0) at 95 °C for 15 min and subsequently cooled down at RT. Two washes were performed with Tris-buffered saline 0.1% Triton X-100 (TBS-T), then samples were blocked with TBS-T 3% Normal Goat Serum (NGS) and 1% BSA for 2 h, and finally incubated overnight at 4 °C with 10 µg/mL of anti-Amhr2 primary antibody [52] in TBS-T 3% NGS and 1% BSA. The day after, sections were washed twice in TBS-T, immersed in TBS 0.5% hydrogen peroxide for 20 min to quench endogenous peroxidase activity, and then incubated for 1.5 h at room temperature with secondary antibody (goat anti-rabbit IgG- HRP conjugated) (GAR-HRP, Bio-Rad Laboratories, Inc., Hercules, CA, USA) diluted 1:200 in TBS-T 3% NGS, 1% BSA. After three washes in TBS-T, slides were treated during 3–5 min with 3,3′-Diaminobenzidine (DAB, ACROS Organics, Waltham, MA, USA) used as substrate for colour development. Nuclei were counterstained with 25% hematoxylin (Sigma-Aldrich^®^, St. Louis, MO, USA) for 10 s. Slides incubated without primary antibody served as a negative control. Sections were examined and photographed by a Nikon Eclipse E600 imager microscope (Nikon Instruments, Europe BV, Kingston, Surrey, England).

### 4.4. In Vitro Testis Culture

Three different *in vitro* experiments were performed using testes from adults of two different species, European sea bass and medaka.

Testes from adult European sea bass in the pre-meiotic stage were collected in September–October and processed as described previously [62]. After fish dissection, gonad tissue was removed and immersed in ice-cold Dissection Sea Bass Ringer (DSBR, consisting of 130 mM NaCl; 5 mM KCl; 1 mM Na_2_HPO_4_; 25 mM Hepes, 5 mM Glocose, titrated to 7.4 with NaOH) containing 0.5% Bovine Serum Albumin (BSA, fraction V, Sigma-Aldrich^®^, St. Louis, MO, USA), 100 U/mL Penicillin/Streptomycin (Pen/Strep, Life Technologies, Inc., Life Technologies™ Ltd., Paisley, Scotland, UK) and 100 µg/mL Geneticin (G-418 sulphate, Life Technologies, Inc., Life Technologies™ Ltd., Paisley, Scotland, UK). Then, testes, maintained on ice, were thoroughly chopped into small fragments with a razor blade until forming a paste. After repeated washes on DSBR and centrifugation (60× *g*, 10 min, 4 °C), pieces of the gonadal preparation were transferred to 96-well plates (about 15–20 mg per well), with 0.1 mL of Sea Bass Ringer (SBR; according to Sorbera et al. [103]) containing 0.5% BSA, 100 U/mL Pen/Strep, and 100 µg/mL G-418, and pre-incubated for 1 h at 21 °C under shaking conditions (100 rpm). Then, the explants were subjected to different treatments, each one performed in triplicate. Each experiment was repeated with 5 to 6 different animals. The medium was replaced by 100 µL of fresh SBR containing the indicated concentrations of *P. pastoris* or CHO-derived recombinant sbAmh or sb-scFsh. As controls, SBR or sham culture media from *P. pastoris* or CHO cells were used. After 24 h of incubation, the medium was collected and stored at −20 °C until steroid analysis. The gonadal explants were deep-frozen in liquid nitrogen and stored at −80 °C until RNA extraction. In some experiments the cAMP-pathway competitive inhibitor Rp-8-CPT-cAMPS was used. In these cases, testis explants were pre-incubated with Rp-8-CPT-cAMPS for 1 h just before adding treatments with the corresponding hormones or control media.

In vitro culture was also performed by using testes from adult Japanese medaka (*Oryzias latipes*) (N = 43). Explant preparation was carried out as described above. The tissue was not distributed into wells based on weight due to the very small size of the gonad. For each animal, the two testis fragments were separated and each one was placed in a well. One half was treated with 1 µg/mL of sbAmh produced by CHO cells, and the other half from the same animal was treated with CHO control medium. After 24 h of incubation, medium and testis pieces were collected and stored for posterior analyses, as described above.

### 4.5. Injection of sbAmh Plasmid In Vivo

Injection of pcDNA3-sbAmh (sbAmh group) and empty pcDNA3 (control group) expression plasmids was performed following the protocol already established in our previous works [46,104]. Plasmids were prepared using an anion-exchange resin (Plasmid Mega Kit; Qiagen, Hilden, Germany). Fish were anesthetized in MS-222 (0.1 mg/L water) and injected intramuscularly to a depth of 4–6 mm in the left epaxial muscle anterior to the dorsal fin using an insulin syringe and a 26.5-gauge needle. To ensure delivery of the plasmids to the recipient cells, DNA injection was immediately followed by four electroporation pulses of 90 V/cm and 20 msec in the same area of the fish’s body, using needle-array-type electrodes (2-Needle Array Model 531; BTX, Holliston, MA, USA) and a ECM830 (BTX, Holliston, MA, USA) generator. The experiment was performed in summer (July–August) with 2- and 4-year-old sea bass with pre-meiotic testes (N = 33; weight, 741.76 ± 58.87 g [mean *±* SEM]). The control (N = 13) and sbAmh (N = 15) groups underwent two injections, on days 0 and 3, with 100 µg of plasmid/animal in a final volume of 100 µL of PBS. On day 0, a group of non-injected fish (N = 5) was euthanized to evaluate initial gonadosomatic index (GSI) and gonad developmental stage by histological analysis. All injected fish were anesthetized to obtain blood samples for sex steroid analysis on days 0, 14, 21, 31. On day 14, halfway through the experiment, 6 fish per group were sacrificed. The remaining animals were sacrificed on day 31, at the end of the experiment. In all cases, the gonads were dissected for GSI evaluation, histological analysis, and RNA extraction.

### 4.6. Sex Steroid Analysis

Testosterone (T) and 11-ketotestosterone (11-KT) contents in the culture medium of testes and in the plasma of injected fish were evaluated by enzyme immunoassays (EIAs) previously validated for their use in sea bass in our laboratory [79,105]. First, steroids were extracted from the culture medium or from plasma with methanol, then the organic solvent was evaporated and the dry extract visible as a pellet was reconstituted in assay buffer (EIA buffer, consisting of 100 mM Potassium Phosphate Buffer PPB; 1.54 mM NaN_3_; 0.4 M NaCl; 1 mM EDTA) containing 0.1% Bovine Serum Albumin (BSA, fraction V, Sigma-Aldrich^®^, St. Louis, MO, USA). The assays were performed in a final volume of 150 µL/well, in duplicate, in 96-well microtiter plates previously coated with mouse anti-rabbit IgG monoclonal antibodies (Clone RG-16, Sigma-Aldrich^®^, St. Louis, MO, USA).

Optical density was detected at 405 nm with a microplate reader (TECAN Infinite M Plex, Männedorf, Switzerland). For the evaluation of T contents, the sensitivity (B_i_/B_0_ = 90%) and the half-displacement (B_i_/B_0_ = 50%) of the enzyme immunoassays ranged between 5 and 8 pg/mL and 59 and 113 pg/mL, respectively. For 11-KT contents, they ranged between 0.5 and 1 pg/mL and 6 and 13 pg/mL, respectively.

### 4.7. RNA Extraction, Reverse Transcription, and Quantitative Real-Time PCR (qPCR)

The gene expression of *hsd3b*, *amhr2*, *ar*, *cyp11b*, and *cyp17a1* in testis explants cultured *in vitro* was determined by quantitative real-time PCR (RT-qPCR). Total RNA was extracted from 15 to 20 mg of tissue, previously homogenized by Savant FastPrep FP120 (Cambridge Scientific, Watertown, MA, USA), using the Maxwell™ 16 LEV simplyRNA Tissue Kit (Promega Corp., Madison, WI, USA) on a Maxwell™ 16 Instrument (Promega Corp.). The absence of genomic DNA was checked by performing a control PCR directly on the RNA samples. For cDNA synthesis 1 µg of RNA was reverse-transcribed using Superscript IV (Invitrogen Corp., Carlsbad, CA, USA) with random hexamers as primers, following the manufacturer’s instructions. Ribosomal protein L13a gene (*rpl13a*) was used as endogenous reference gene for calibration [106]. Samples were analyzed in duplicate, and qPCR assays were performed on 96-well plates and run in a CFX384 Touch™ Real-Time PCR Detection System (Bio-Rad Laboratories, Inc., Hercules, CA, USA) using default settings for each fluorescence detection system. The optimized amount of primers and probes and the cDNA sample dilution used for each gene assay are shown in Table S1 [51,58,106,107]. All qPCR components for the target genes or reference gene were mixed with 5× PyroTaq PROBE qPCR Mix Plus No-ROX (CMB-Bioline, Madrid, Spain) or 5× PyroTaq EvaGreen Mix Plus No-ROX (CMB-Bioline, Madrid, Spain), respectively, up to a final reaction volume of 20 μL. To correct for variability in amplification efficiency between different cDNAs and to determine the gene expression levels in the *hsd3b*, *amhr2*, *ar*, *cyp17a1*, and *rpl13a* qPCR assays, appropriate standard curves were used, consisting of tenfold serial dilutions of known concentrations of plasmids containing these target genes. In the case of *cyp11b*, the relative expression was calculated by the ΔCt method, in comparison with housekeeping ΔCt values, due to the unavailability of the respective plasmid for a standard curve. Data were captured and analyzed with CFX Manager™ Software (version 4.1). The correlation coefficients (R^2^) of the standard curves ranged from 0.866 to 0.999 and PCR efficiencies ranged from 75.7 to 109.1%. In the case of the EvaGreen assay, the melting curve generated a single peak, confirming primer specificity.

### 4.8. Cell Culture, Transfection, and Luciferase Assay

The expression plasmid for sea bass Amhr2 (pcDNA3-sbAmhr2) was available from a previous study [51] and the one for human AMHR2 was a gift from Dr. Nathalie di Clemente. The BRE-Luc [108] and CRE-Luc (BD Clontech, Palo Alto, CA, USA) reporter plasmids contain the luciferase gene under the control of BMP (BRE)- or cAMP (CRE)-responsive elements, respectively. The pEGFP plasmid (Promega, Corp., Madison, WI, USA) constitutively expresses EGFP and was used to check transfection efficiency in all cases.

African green monkey kidney fibroblast-like cells (COS-7) were maintained in Dulbecco’s modified eagle medium (DMEM) GlutaMAX (Life Technologies, Inc., Life Technologies™ Ltd., Paisley, Scotland, UK) supplemented with 10% Fetal Bovine Serum (FBS), and 100 U/mL Penicillin/Streptomycin (Pen/Strep, Life Technologies, Inc., Life Technologies™ Ltd.) at 37 °C in a humidified 5% CO_2_ incubator. Cells were seeded in 6-well plates (~0.6 × 10^6^ cells per well), grown to 75–80% confluence, and transiently co-transfected with 2.5 µg of total DNA using Lipofectamine 3000 Reagent (Invitrogen Corp., Carlsbad, CA, USA) according to the manufacturer’s protocol (final volume of transfection 250 µL/well). The plasmid DNA proportions in the transfections were as follows: 20% pBRE-Luc reporter plasmid DNA and 80% receptor plasmid (sbAmhr2 or hAMHR2) when analyzing the SMAD pathway, and 50% pCRE-Luc DNA and 50% receptor plasmids (sbAmhr2 or hAMHR2) when studying the cAMP pathway.

The day after the transfections, the medium was replaced, and cells were plated in 96-well plates. After 24 h, cells were treated with hAMH, sbAmh, or 8-Br-cAMP at the indicated concentrations for an additional 24 h in DMEM with 1% FBS and 100 U/mL Pen/Strep. Control treatments consisted of addition to the culture media of the corresponding volumes of hAMH resuspension buffer (4 mM HCl, 0.1% BSA in PBS) as control for hAMH or culture media from empty pcDNA3-transfected CHO cells as control for sbAmh. DMEM 1%FBS was used as a reference for standard culture conditions. In some experiments the signalling pathway inhibitors LDN-193189 hydrochloride and Rp-8-CPT-cAMPS were used for inhibition of SMAD and cAMP pathways, respectively. These inhibitors were added to the culture medium 1 h before sbAmh treatment in order to guarantee the inhibitory action. When Rp-8-CPT-cAMPS was used in CRE-Luc activation trials, the subsequent stimulatory treatment was performed for 4 h, instead of 24 h, as this inhibitor loses effectivity over time. All treatments were tested in triplicate and each assay was performed at least three times. After incubation, cells were washed with PBS to remove phenol red (which interferes with detection) and lysed in 20 µL/well of 1× Passive Reporter Lysis Buffer (Promega Corp., Madison, WI, USA). Luciferase activity was determined by mixing the lysed cells with 100 µL of luciferin reagent (20 mM Tricine KOH, pH 7.8; 0.1 mM EDTA; 8 mM MgCl_2_; 33.3 mM dithiothreitol (DTT); 270 µM coenzyme A; 530 µM ATP; 400 µM luciferin) and measuring the light emitted with a luminometer (Junior LB 9509 Portable Luminometer, Berthold Technologies GmbH & Co. KG; Bad Wildbad, Germany) in relative light units (RLU). The results were normalized by the DMEM treatment and expressed as fold inductions of Firefly luciferase with respect to the control treatment of each assay.

### 4.9. Statistical Analysis

Data are shown as the mean ± standard error of mean (SEM), and all statistical analysis and data representations were performed by using GraphPad Prism version 8.1 (GraphPad Software, Inc., La Jolla, CA, USA). One-way ANOVA followed by post hoc Tukey’s multiple comparison test was used to determine significant differences in (i) steroid content in the culture medium of European sea bass testis (Figure 2A–D); (ii) gene expression in the cultured tissue (Figure 3); (iii) luciferase activity within stimulatory groups in COS-7 transactivation assays (Figure 6). The Mann–Whitney U test was used to analyze differences in steroid content in the culture medium of Japanese medaka (Figure 2E,F), whilst Student’s *t*-test was performed to compare luciferase activity between stimulatory and control treatments in the COS-7 transactivation assays (Figure 5 and Figure 6). Two-way ANOVA, followed by post hoc Tukey’s or Sidak’s multiple comparison test, allowed us to determine statistical differences in steroid levels in the culture medium of European sea bass testes treated with an inhibitor (Figure 7). In all tests, differences were accepted as statistically significant starting from *p* < 0.05. The Kruskal–Wallis non-parametric test (ANOVA on ranks), followed by post hoc Dunn’s multiple comparison test, and the Mann–Whitney U test were used to account for differences between groups in steroid plasma levels of injected fish because normality requirements were not fulfilled (Figure 4).

## 5. Conclusions

Our findings in the pre-meiotic testis of European sea bass provide the first evidence in non-mammalian vertebrates that Anti-Müllerian hormone exerts a steroidogenic function through the cAMP/PKA signalling pathway mediated by its interaction with the specific type II receptor Amhr2. The consistent results obtained in medaka testis culture suggest that the steroidogenic action of Amh is not a peculiarity restricted to the European sea bass, but rather a more widespread mechanism among teleost fish. The observed crosstalk between the canonical Smad pathway and the non-canonical cAMP/PKA signalling route, with the key involvement of PKA in promoting steroid production, reveals a novel mechanism of Amh action, previously, to our knowledge, unreported in teleosts. This dual signalling capacity may underpin the steroidogenic effects of Amh and offers new insight into its multifaceted role in fish reproductive physiology. These findings open new avenues for investigating the molecular mechanisms by which Amh regulates steroidogenesis and warrants further research to clarify its broader physiological relevance.

## Data Availability

Data are available on request due to restrictions, e.g., privacy or ethical.

## Supplementary Materials

The following supporting information can be downloaded at: https://www.mdpi.com/article/10.3390/ijms26157554/s1.

## Abbreviations

The following abbreviations are used in this manuscript:

11-KT	11-ketotestosterone
ALK	Activin receptor-like kinase
AMH	Anti-Müllerian hormone
Amhr2	Anti-Müllerian hormone type II receptor
ATP	Adenosine triphosphate
BPG	Brain–pituitary–gonad axis
BRE	BMP response element
BSA	Bovine serum albumin
cAMP	Cyclic adenosine monophosphate
CHO	Chinese hamster ovary
COS-7	Cell line from African green monkey kidney fibroblasts
CRE	cAMP response element
CREB	cAMP response element-binding protein
DAB	3,3′-Diaminobenzidine
DMEM	Dulbecco’s modified eagle medium
DMSO	dimethyl sulfoxide
DSBR/SBR	(Dissection) sea bass ringer
E2	17β-estradiol
EGFP	Enhanced green fluorescent protein
EIA	Enzyme immunoassay
FBS	Fetal bovine serum
Fshr	Follicle-stimulating hormone receptor
FSH	Follicle-stimulating hormone
GAR-HRP	Goat anti-rabbit horseradish peroxidase
GnRH	Gonadotropin-releasing hormone
GSI	Gonadosomatic index
hAMH	Human anti-Müllerian hormone
hAMHR2	Human anti-Müllerian hormone type II receptor
LH	Luteinizing hormone
NGS	Normal goat serum
PBS	Phosphate-buffered saline
PFA	Paraformaldehyde
PGCs	Primordial germ cells
PKA	Protein kinase A
PKA-C	Protein kinase A catalytic subunit
PKA-R	Protein kinase A regulatory subunit
pPIC9K	*Pichia pastoris* expression vector pPIC9K
RLU	Relative light units
sbAmh	Sea bass anti-Müllerian hormone
sb-scFsh	Sea bass single-chain follicle-stimulating hormone
SCF	Stem cell factor
SMAD	Small mother against decapentaplegic
T	Testosterone
TBS/TBS-T	Tris-buffered saline with Triton X-100
TFG-β	Transforming growth factor beta
