# Supplementary material for: A Novel Steroidogenic Action of Anti-Müllerian Hormone in Teleosts: Evidence from the European Sea Bass Male (Dicentrarchus labrax)
Source: Int J Mol Sci. 2025 Aug 5;26(15):7554. doi: 10.3390/ijms26157554 (PMC12347443; doi:10.3390/ijms26157554)
Supplement: Supplementary file 1 [file ijms-26-07554-s001.zip › ijms-3759129-supplementary.pdf]

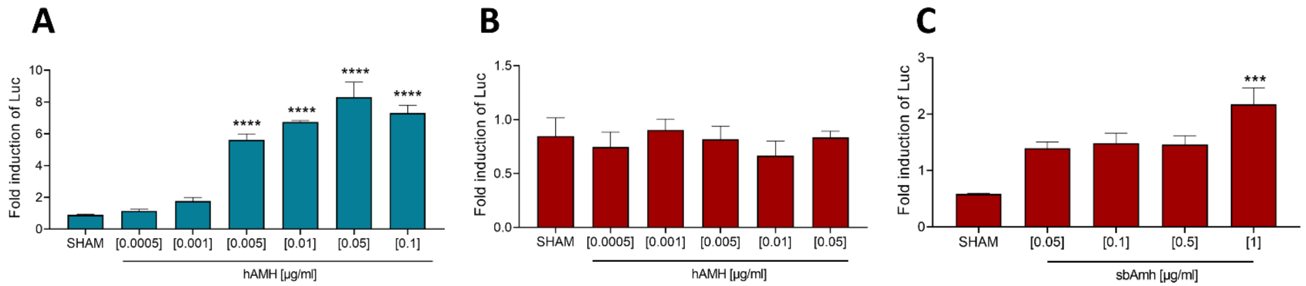

Figure S1. Human AMH (hAMH) and recombinant sea bass Amh (sbAmh) actions on the Smad (BRE-Luc) and cAMP (CRE-Luc) signalling pathways tested in COS-7 cells. hAMH induction of (**A**) BRE-Luc and (**B**) CRE-Luc reporter activities through the specific receptor hAMHR2. (**C**) sbAmh induction of CRE-Luc reporter activity through the specific receptor sbAmhr2. Firefly luciferase activities were measured as relative light units (RLUs), and expressed as fold induction respect to the reference control 1% FBS DMEM treatment. All treatments were tested in triplicate and each assay was performed at least three times. Data are reported as *mean ± standard error of mean* (SEM) and analysed by the one-way ANOVA followed by post-hoc Tukey's test. Asterisks represent statistical differences (* $p<0.05$; ** $p<0.01$; *** $p<0.005$; **** $p<0.001$) of the treatment compared to its control. SHAM: (**A-B**) 1% FBS DMEM with hAMH resuspension buffer (4mM HCl, 0.1% BSA in PBS); (**C**) CHO cells sham culture medium.

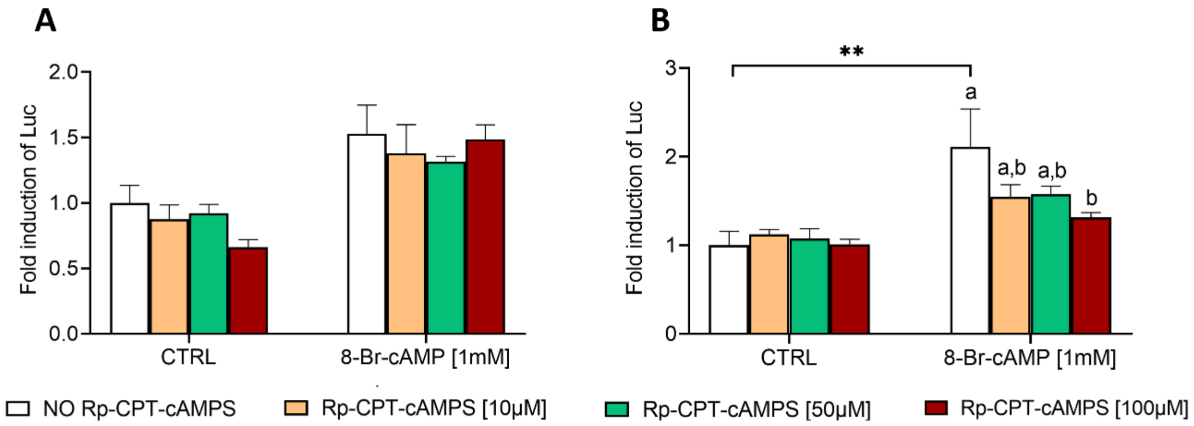

Figure S2. Rp-CPT-cAMP effect on the cAMP signalling pathway stimulated by 8-Br-cAMP tested in COS-7. 8-Br-cAMP induction of CRE-Luc reporter activity after (**A**) 24h treatment or (**B**) 4h treatment. Firefly luciferase activities were measured as relative light units (RLUs), and expressed as fold induction respect to the control treatment (CTRL: 1% FBS DMEM without Rp-CPT-cAMPS, set to 1). All treatments were tested in triplicate and each assay was performed at least three times. Data are reported as *mean ± standard*

error of mean (SEM) and analysed by the one-way ANOVA followed by post-hoc Tukey's test within the stimulatory group, by Student's t.-test between stimulatory treatment and control treatment in absence of inhibitor. Letters indicated statistical differences (p<0.05) among treatments in the same stimulatory group. Asterisks represent statistical significance (* p<0.05; ** p<0.01; *** p<0.005; **** p<0.001) between stimulatory and control treatments in absence of inhibitor.

Table S1. List of analysed genes, primers and probes used for RT-qPCR.

Gene	Sequence (5′ → 3′) [a]	nM [c]	Amplicon size	qPCR efficiency	cDNA dilution	First published
hsd3b (JQ861952) [b]	fw: AGG TGA AGG GTG GAC AGT GTT T	900	66 bp	75.6%	1/1	[107]
	rv: TGG CCC TCG CCA TAG ATC	900				
	pr: [6~FAM]CAC ACA TGC TCT CTG AGG CCG ACG[TAMRA]	125				
amhr2 (JQ801443.1) [b]	fw: CCATCCTGCGTTCTTGTTCA	300	67 bp	100%	1/2	[51]
	rv: TGAGCAAGACCCATGTTTGC	300				
	pr: [6~FAM]AATCGCCACTGGTCGAGCCACAC[TAMRA]	125				
ar (AY647256.1) [b]	fw: CGG CTG AGG AAG TGT TTT GAA	300	74 bp	96.8%	1/1	[107]
	rv: GTT TTT CTG TTG TCC AAT CTT CTT TAG TT	300				
	pr: [6~FAM]CCG GAA TGA CTC TCG GAG CAC GC[TAMRA]	300				
cyp11b1 (AF449173) [b]	fw: CCT GTT GCT CCG TGT TCG T	300	66 bp		1/2	[58]
	rv: CTG AAG ATG TGA TCC CAT GCA	900				
	pr: [6 ~ FAM]CCT CTG TGG ACC AAG CAC GCC A[TAMRA]	100				
cyp17a1 (JQ861953) [b]	fw: GCC GTC ACC AAT GTC ATC TG	300	77 bp	104%	1/1	[107]
	rv: CAG CAT GGC CTC GAA CTC A	50				
	pr: [6 ~ FAM]TCA CTG TGC TTC AAC TCG TCC TAC CGC[TAMRA]	125				
rpl13a (DT044539) [b]	fw: TCTGGAGGACTGTCAGGGGCATGC	100	148 bp	93.9%	1/50	[106]
	rv: AGACGCACAATCTTGAGAGCAG	100				

[a] Forward (fw) and reverse (rv) primers were obtained from Invitrogen™ (Life Technologies). Hydrolysis probe (pr) was purchased from Eurofins Genomics, Germany.
[b] GenBank accession no. for sea bass gene.
[c] Amount of primer or probe in the PCR.
